# The Review of the Autotomy of Agamid Lizards with Considerations about the Types of Autotomy and Regeneration

**DOI:** 10.3390/jdb9030032

**Published:** 2021-08-16

**Authors:** Natalia Borisovna Ananjeva, Dmitry Anatolyevich Gordeev, Dmitry Vyacheslavovich Korost

**Affiliations:** 1 Zoological Institute of RAS, 199034 St. Petersburg, Russia; 2 Institute of Natural Sciences, Department of Biology, Volgograd State University, 400062 Volgograd, Russia; dmitriy8484@bk.ru; 3 Russian Federal Research Institute of Fisheries and Oceanography (VolgogradNIRO), 400001 Volgograd, Russia; 4 Department of Geology and Geochemistry of Fossil Fuels, Faculty of Geology, Lomonosov Moscow State University, 119991 Moscow, Russia; dkorost@mail.ru

**Keywords:** Squamata, Agamidae, Ophidia, caudal autotomy, urotomy, evolution

## Abstract

We present a review of the data on the intervertebral autotomy and regeneration of agamid lizards based on an analysis of information obtained over a 35-year period after the publication of thorough reviews (Arnold, 1984, 1988 and Bellairs, Bryant, 1985). It is supplemented by our own studies of 869 specimens of agamid lizards (Sauria, Agamidae) stored in the herpetological collections of the Zoological Institute of the Russian Academy of Sciences (St. Petersburg, Russia) and the Zoological Museum of the Moscow State University (Moscow, Russia), represented by 31 species of 16 genera. The manifestations of the ability for autotomy and regeneration in phylogenetic lineages within the family—Leiolepidinae, Amphibolurinae, Agaminae, Draconinae—are considered. A comparative morphological analysis of the structure of the caudal vertebrae was carried out using the Computer Microtomography Methods (micro-CT) in the following ecomorphological types of agama: (1) with developed abilities to caudal autotomy and regeneration, (2) with the ability to caudal autotomy but without regeneration and (3) without the ability to autotomy. The phenomenon of intervertebral autotomy (urotomy) in snakes is considered too. Possible ways of evolution of the ability to caudal autotomy as a defense strategy against predators are discussed in the phylogenetic context.

## 1. Introduction

Fredericq [1], who described the reflex severance of an appendage, originally introduced the term autotomy. This phenomenon is widespread in the Animalia kingdom and is characteristic, for example, of some mollusks, echinoderms [2], many arthropods [3] and some mammals, for example, *Sigmodon hispidus* [4]. In vertebrates, cases of autotomy can be found in amphibians and reptiles. In these animals, it is expressed to varying degrees in the form of tail autotomy (or caudal autotomy) in some Caudata and among Lepidosauria in tuatara, many lizard species, a number of amphisbaenians and some snakes [2,5,6,7,8,9,10,11,12]. Among recent reptiles, caudal autotomy has been unknown only for Testudines and Crocodilia [6]. Finally, occasional cases of tail regeneration have been observed in crocodilians (crocodiles, alligator, caiman [2,13]). The new tail can measure over 40 cm in length and the segmented vertebrae of the amputated tail are replaced with a calcified rod of cartilage. The frequency of this phenomenon in crocodilians is not exactly known, but it seems to be very low. Recent studies have shown regenerative activity and tail recovery up to 6–18% of total body length in *Alligator mississippiensis* [14]. The caudal vertebrae were replaced by a ventrally located non-segmented endoskeleton and skeletal musculature was absent, instead consisting of fibrous connective tissue composed of type I and type III collagen fibers. The histological aspects of this process are not known but it probably occurs such as the process that takes place in the lizard ablation tail, but over a much longer period. In some cases of extensive—but not lethal—injury to the maxilla, a large portion of the skeletal tissue is regenerated as cartilaginous tissue over a period of 2–3 years [15]. In general, in vertebrates the power of regeneration decreases from Urodela to larval Anura and fish, then to adult Anura and reptiles (lizards), and, finally, birds and mammals, where regeneration is almost absent [10]. The living fossil *Sphenodon punctatus* (Sphenodontidae, Lepidosauria), a lizard-like reptile presently living on a few offshore islands of New Zealand, has a regenerative power similar to that of agamid lizards [10,16].

The issue of caudal autotomy has several interrelated, interdisciplinary aspects. The study of autotomy and regeneration processes includes many fields of modern biology, from paleontology to biochemistry. A deep interest in these phenomena appeared in the last third of the twentieth century, when extensive and detailed reviews were published about the structural features of autotomizing tissues and their morphogenesis during regeneration [2], and an ecological aspect which considers autotomy as an adaptation [6]. A comparative analysis of the incidences of autotomy in different families of squamate reptiles seems to be especially interesting both for a deeper understanding of strategies for avoiding predators and for clarifying the existing ideas about the phylogeny of reptiles in general [5].

Reptile caudal autotomy, in its “classic” form, is normally a tail fracture at specific locations, commonly known as fracture planes or autotomy planes. Such morphology determines the special adaptations of various tissues of the tail and, first of all, of the vertebrae. As a rule, each vertebra is more or less split at the sites of the fracture plane. In most lizards capable of autotomy, a fracture plane is recorded in every caudal vertebra in the so-called pygal series.

Among lepidosaurian reptiles, autotomy occurs in Rhynchocephalia (Sphenodontidae: *Sphenodon punctatus*) and in its sister group, Squamata (Sauria, Ophidia and Amphisbaenia) [2,16]. This phenomenon in Sauria is typical for most species of superfamilies Gekkota (Gekkonidae, Diplodactylidae, Eublepharidae, Phyllodactylidae, Pygopodidae, Sphaerodactylidae), Iguania (Iguanidae), Scincomorpha (Scincidae, Lacertidae, Cordylidae, Gerrhosauridae, Xantusiidae), Gymnophthalmoidea (Teiidae, Gymnophthalmidae, Alopoglossidae), Diploglossa (Anguidae) and Dibamia (Dibamidae). The cartilaginous layers between the two bony halves are absent in the vertebrae of Acrodonta Iguania—chameleons (family Chamaeleonidae) and agamids (family Agamidae)—, as well as all Platynota (Varanidae, Lanthonotidae, Helodermatidae). More recently, autotomy and partial regeneration have been confirmed in the superfamily Shinisauroidea (Shinisauridae) [5] (own data [17]).

The caudal autotomy is often accompanied by its subsequent regeneration. This process is similar in a number of fundamental features to other regenerative processes in vertebrates. It includes wound healing, blastema formation, differentiation and growth. It is known that some lizards are capable of imperfect regeneration of limbs [2,10,18], from regenerative buds to tail-like outgrowths. The regenerative abilities of tissues, preserved in the evolutionary history of reptiles, are expressed in the regeneration of the autotomized tail. The regenerated tail differs from the original: a cartilaginous tube, which is usually calcified, replaces the bony vertebrae; the location of muscle fibers changes, the fat layer often increases and the normal tail pholidosis is disrupted, which was considered by Boulenger [19] as a primitive condition for this group. Most information about reptilian tissue regeneration derives from the study of the regenerating tail of lizards, an organ that measures a few centimeters in length in most of the species studied. A few other examples of organ regeneration among reptiles include the regeneration of the tail and jaws of crocodilians and the shell of turtles [2,10,18]. Fracture planes are registered in the lepidosaurian the most and can be considered as a common primitive rhynchocephalian and saurian character [2]. The tail of tuatara breaks by intravertebral autotomy; this is usually, but irregularly, followed by the ablation of a terminal piece of vertebra [16]. These authors favor the hypothesis that tail autotomy in *Sphenodon* is imperfect due to remaining at an early evolutionary stage.

Earlier [2,5,6], two ways of autotomy as defense against predators were described for reptiles: intravertebral with rupture occurs through the vertebral body at “weak sites” occurring in “places of weakness” through the vertebral body containing the cartilaginous plate; in the less common intervertebral autotomy, the break planes are absent and the tail breakage occurs between adjacent vertebrae. However, further studies of the morphology of the tails of different reptile taxa, including snakes, and the accumulation of information on autotomy in other groups (Amphibia, Mammalia) led to the need to revise and expand this terminology. According to the more new proposed terminology [8,11], the following types of breaks have been distinguished: urotomy (any type, both inter- and intravertebral), autotomy per se (which, according to the authors, include only the intravertebral-type of break with the subsequent regeneration of the lost part) and pseudoautotomy (an intervertebral nonspontaneous type of tail fracture without regeneration). Intervertebral autotomy was reliably noted in a number of the agamid lizards, as well as in some snakes belonging to three families: Colubridae, Lamprophiidae and Natricidae [12,20]. In this classification, agamid lizards are not assigned to any of the above categories; they occupied an indefinite position (“intermediate condition”) [11,21]. Intervertebral autotomy in snakes is not accompanied by a subsequent regeneration in contrast to some genera of agamid lizards.

One of the most interesting groups for the study of urotomy is iguanomorphic lizards (Iguania), since it includes species with all known ways of autotomic tissue rupture or that have lost this ability. Iguania is a diverse group that includes about 2000 extant species and is probably terminal in relation to the rest of Sauria [22], which makes them a convenient object for understanding the evolution of urotomy in Lepidosauria. Agamidae is a family within Iguania which includes species with or without the ability to autotomy and regenerative development [8]. In some genera of agamid lizards, the formed regenerates can have an unusual shape, which sharply differs from the original tail [20,23].

At the end of the 19th century, F. Siebenrock [24] was probably the first who noted that the autotomy and regeneration occur in agamids, and followed by the regeneration and the formation of a cartilaginous tube at the site of the fracture. He recorded these facts in draconine agamids *Gonocephalus* (*Coryphophylax*) *subcristatus*, *Calotes mystaceus*, *C. ophiomachus* (*C. calotes*), agamine agamids *Agama* (*Laudakia*) *tuberculata*, *A.* (*Paralaudakia*) *himalayana* and amphibolurine agamids *Amphibolurus* (*Ctenophorus*) *decresii.* According to modern views [25], they belong to three subfamilies. Siebenrock as a morphologist provided a detailed description of all elements of the skeleton of agamids lizards, including the caudal part of vertebral spine, based on the examination of specimens from the Naturhistorisches Museum Wien (Natural History Museum Vienna) collections. Afterwards, all the details of breakage and regeneration frequencies of agamids based on the examination of the specimens stored in the British Museum (Natural History) were given in the fundamental review by Arnold [5].

Due to the accumulation of a large amount of data, a revision of the taxonomy of many groups within this family, as well as a revision of ideas about the phylogeny of squamate reptiles [22,26,27,28,29,30], it became necessary to reconsider the evolution of the ability to autotomy in Agamidae. Therefore, this is the main goal of the present manuscript.

## 2. Materials and Methods

The material for this study was newly collected data on the autotomy and regeneration based on study of specimens from the herpetological collections of the Zoological Institute, St. Petersburg, Russian Academy of Sciences (ZISP RAS, St. Petersburg, Russia), and the Zoological Museum, Moscow State University (ZMMSU, Moscow, Russia). These data were combined with known literature for different aspects of tail shedding in agamid lizards, including osteological data of specimens from the collection of the Natural History Museum Vienna (Naturhistorisches Museum Wien) [24] and British Museum (Natural History) [5]. We examined a total of 998 museum specimens of 29 species belonging to 15 genera. For some specimens, it was difficult to reveal the fact of autotomy, so they were excluded from the study. Thus, the actual sample was 868 specimens (Table 1).

To analyze the localization of autotomy, the following classification was used (Arribas, 2014): intact tail, pseudoautotomy in the distal third, in the middle and in the proximal third of the tail. The anatomical structure of the tails was studied by the micro-CT method (Moscow State University, Moscow, Russia) using a SkyScan-1172 desktop scanner (Bruker microCT, Kontich, Belgium) equipped with a Hamamatsu 10 Mp digital camera. The tails of *Paralaudakia caucasia* (ZISP 31550), *Calotes versicolor* (ZISP 14261), *Laudakia nupta* (ZISP 11397.5), *Mantheyus phuwuanensis* (ZISP 30558), *Intellagama lesueurii* (ZISP 20474) and *Trapelus sanguinolentus* (ZISP 31548) and for comparison of *Lacerta agilis* ZISP 31549 were scanned. The tail samples were placed in a plastic vessel and scanned sequentially. Scanning was performed with a resolution of 6.19 μm at a source voltage of 40 kV and current strength of 250 μA with a turn step of 0.4° and a shutter speed of 110 ms. The resulting subscan data arrays were connected vertically to obtain the general tomogram. Data were processed using the SkyScan software DataViewer, CTAn, and CTVol (creation and visualization of 3D models).

An X-ray study of the autotomized tail of *Paralaudakia caucasia* was carried out on the equipment of the ZISP RAS (St. Petersburg, Russia).

## 3. Results and Discussions

Most of the specimens studied had intact tails (Table 2), except for series represented by a small number of specimens. In such cases, the number of specimens with intact tails was less than 40.0%. If urotomy was found, it was localized, as a rule, to the distal part (10.5–100.0% of the total sample size), less often in the middle or proximal third of the tail. Because the samples differed greatly in size (from 1 to 237 specimens depending on the species), a statistical analysis of these data was impossible.

Caudal autotomy has traditionally been considered as a defensive strategy in many lizards and some snakes. An important aspect for understanding the role of autotomy and pseudoautotomy is recording the place of rupture, which depends on a number of factors. Firstly, this is associated with the tissue rupture in some particular area: there are pygal or non-autotomic vertebrae and postpygal or autotomic vertebrae located behind them. The shedding of the tail in agamids can take place between the postpygal vertebrae only. Musculature participating in a reproductive function and locomotion is attached to the pygal vertebrae; therefore, its damage is biologically disadvantageous. Secondly, it depends on the area of the tail that has been caught by a predator or a conspecific lizard. It was noted that autotomic tissue rupture always occurs only slightly above the capture site and is explained by energy savings for the subsequent regeneration of the appendage, preservation of the partial function of the tail as a balancer, etc., if the species is capable of regeneration [31].

In those species that are able to lose the tail, a pseudoautotomy was recorded, which mostly confirms the earlier literature data, but we also added some new data on several taxa (Table 2). The fact of pseudoautotomy was recognized if a regenerate was formed in vivo or the wound was healed in vivo. Of the 29 species studied by us, this type of urotomy is typical for some *Leiolepis* Cuvier, 1829: *L. belliana*, *L. guentherpetersi*, *L. guttata*, *L. reevesii*, *Intellagama lesueurii*, *Lophosaurus spinipes*, *Physignathus cocincinus*; *Laudakia* Gray, 1845: *L. nupta* and *L. tuberculata*; *Paralaudakia* Baig, Wagner, Ananjeva and Böhme, 2012: *P. caucasia*, *P. erythrogaster*, *P. himalayana*, *P. lehmanni*, *P. microlepis*, *P. stoliczkana*, *Stellagama stellio*; *Calotes* Cuvier, 1817: *C. calotes*, *C. versicolor*; *Gonocephalus* Kaup, 1825: *G. chamaeleontinus*, *G. liogaster*, *Mantheyus phuwuanensis*, *Otocryptis wiegmanni* and *Pelturagonia nigrilabris*. All examined specimens of *Pogona barbata*, *P. vitticeps*, *Gonocephalus sophiae* and *Malayodracon robinsonii* were found to have intact tails. In the literature, we were unable to find any information on the ability of these species to employ pseudoautotomy, and, therefore, the data require further clarification. We can assume that pseudoautotomy could occur in *Gonocephalus sophiae* based on the fact that such autotomy has been recorded in other species of this genus (*G. chamaeleontinus* and *G. liogaster*). The analysis of autotomy frequencies based on the examination of the museum specimens provided only general information and did not reflect the real situation in populations, which should be studied in the wild. Such studies show that autotomy or pseudoautotomy probably occurs in the distal part of the tail in most lizard species. This was confirmed for anoles [32], whereas in five species of the genus *Iberolacerta* Arribas, 1997, the tail was autotomized either in the distal third or in the middle [33].

Autotomy in any form is a very complex phenomenon, an objective study of which requires a carefully developed methodology. To analyze the frequencies of caudal autotomy in different parts of the range of the same species or between several species, it is necessary to take into account the numerous factors determining the ease of autotomy [6]. Among them: (1) the sample size significantly affecting the ratio of autotomy frequencies [9]; (2) characteristics of the behavior of a particular species or even an individual [34]; (3) hunting strategies of predators and their quantitative relationships in the studied populations [6]. Currently, such a unified methodology for studying the frequency of occurrence of autotomy is still absent; there is no general consensus on the minimum sample size, which significantly complicates the analysis and comparison of the results of studies by different authors. The ability to pseudoautotomize is associated not only with the origin and phylogeny of squamate reptiles, but also with the biological effectiveness of the caudal autotomy as a defense. Urotomy has been noted for those species that are able to lose the tail as a life-saving mechanism. On the contrary, if the tail is involved in intraspecific behavioral communication (*Phrynocephalus*), or is actively used for moving in the water, climbing, etc., its loss can be considered as a disadvantage. The loss of the tail may also prevent it being used for defensive purposes other than autotomy. In some agamid lizards, the tail is used as a weapon in intraspecific combat; for example, males of *Agama agama* strike each other about the head with their tails [6]. It was shown that *A. agama* use the tail in social interactions and supposed that males of this species are more likely to form a clubbed regenerate [23]. The negative consequences of its loss will prevail over the benefits. In such species, the propensity for autotomy will be significantly reduced or completely disappear.

The most detailed reviews of the morphological, developmental and cellular aspects of the phenomena of caudal autotomy and regeneration in squamate reptiles have been published by Bellairs, Bryant [2] and Alibardi [10]. They, and other morphologists, postulate that in the great majority of cases, autotomy in reptiles involves the breaking or discarding of the tail (urotomy) at one or more predetermined sites of weakness, which are known as fracture or autotomy planes.

**Figure 1:**
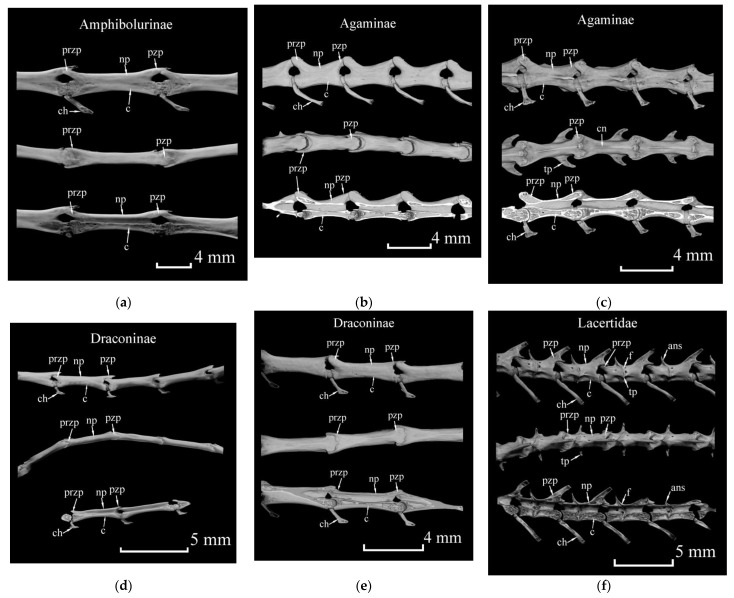
Micro-CT of intact tails of three subfamilies of agamids (with pseudoautotomy or no autotomy) and lacertids (autotomy) with different combinations of caudal autotomy and regeneration. The figure shows (from top to bottom): sagittal plane, frontal plane, a sagittal slice through the middle of the vertebra; (**a**) intact part of tail of *Intellagama lesueurii* (Australia, ZISP 20474), capable of pseudoautotomy and regeneration; (**b**) intact part of tail of *Paralaudakia caucasia* (Russia, ZISP 31550), capable of pseudoautotomy and regeneration; (**c**) intact part of tail of *Trapelus sanguinolentus* (Kazakhstan, ZISP 31548), no autotomy; (**d**) intact part of tail of *Mantheyus phuwuanensis* (Laos, ZISP 30558), capable of pseudoautotomy and regeneration; (**e**) intact part of tail of *Calotes versicolor* (Ceylon (Sri Lanka), ZISP 14261), probably capable of pseudoautotomy without regeneration; (**f**) intact part of tail of *Lacerta agilis* (Russia, ZISP 31549), capable of intravertebral autotomy and regeneration. ans—anterior neural spine; c—centrum; ch—chevron (hemapophysis); cn—crista neurapophysis, neurapophysis crest; np—neurapophysis; przp - prezygapophysis; pzp—postzygapophysis; tp—transverse processes.

Species which are able to autotomize per se which, according to Savage and Slowinski [8] and Costa et al. [11], are characterized by the Intra VB type of break with the subsequent regeneration of the lost part. They have the following morphological characters: intravertebral plane of weakness containing some cartilage cells (Figure 1f) dividing the vertebra into two parts; a well-developed anterior neural region with a vertically oriented process (Figure 1, ans); transverse processes, which may be absent in the distal part of the tail (Figure 1, tp); longitudinal strips of adipose tissue surrounding the vertebral column (absent in the pygal region); segmented muscle tissue, in which myosepta correspond to the plane of fracture. The plane of autotomy delimits these segments along the entire tail, with the exception of the basal, non-autotomic area [2]. The vertebral bodies at the site where the autotomy plane passes are interrupted or become thinner, which facilitates the fracture of the vertebra into two parts; a well-developed anterior neural region with a vertically oriented process (Figure 1, ans); transverse processes, which may be absent in the distal part of the tail (Figure 1, tp); longitudinal stripes of adipose tissue surrounding the vertebral column (absent in the pygal region); segmented muscle tissue, the myosepts of which correspond to the plane of the fracture. Dissimilar to lizards with true autotomy, the postpygal vertebrae of agamid lizards capable of pseudoautotomy contain no cartilaginous region dividing vertebrae into two parts and no vertically oriented processes in the neural regions. Transverse processes are not bifurcated and there is no adipose tissue in the soft tissues in the caudal region.

To reveal the structural features of the caudal vertebrae of Agamidae that vary in their ability for pseudoautotomy and tail regeneration, we studied the postpygal vertebrae of five lizard species from three agamid subfamilies: *Intellagama lesueurii* (Amphibolurinae, pseudoautotomy with regeneration), *Paralaudakia caucasia* (Agaminae, pseudoautotomy with regeneration), *Trapelus sanguinolentus* (Agaminae, non-autotomic species), *Mantheyus phuwuanensis* (Draconinae, pseudoautotomy with regeneration) and *Calotes versicolor* (Draconinae, pseudoautotomy without regeneration). To compare the details of the structure of the vertebrae with pseudoautotomy and real autotomy, a reconstruction of the results of micro-CT of the lacertid lizard *Lacerta agilis* is presented (Figure 1).

The analysis of micro-CT of the postpygal vertebrae showed that non-autotomic agamids (*Trapelus sanguinolentus*) can develop robust postzygapophysis (Figure 1, pzp) with wide articular surfaces that reliably hold adjacent vertebrae and significantly complicate the realization of intervertebral autotomy (Figure 1c), similar to the postzygapophysis of *Lacerta agilis* in which intravertebral autotomy is known (Figure 1f). The postzygapophysis in the caudal vertebra of *Lacerta agilis* firmly connects with the prezygapophysis (Figure 1, przp) of the adjacent vertebra and prevents rupture in the intervertebral region outside the plane of autotomy. The width of the articular surfaces of the postzygapophysis in *Calotes versicolor*, as a species potentially able to autotomy and form a regenerate, was intermediate between *Trapelus* (non-autotomic species) and other agamids with autotomy. In the neurapophysis of *Trapelus sanguinolentus*, ridges were formed for the attachment of the caudal musculature (Figure 1f, cn). This character was absent in other studied agamids capable of losing the tail. The vertebral bodies of the agamid lizards examined, regardless of their ability for pseudoautotomy, were somewhat narrowed in the middle (which does not occur in *Lacerta agilis*, except for a narrow area corresponding to the plane of autotomy), the postzygapophysis was much narrower than in *L. agilis*, the hemapophysis was attached to the articular process of the distal part of the vertebral body. The neurapophysis did not form vertically oriented processes in the central part, which are common in *L. agilis*. Our results showed the longer vertebra bodies in arboreal agamids (Figure 1d,e) in comparison with terrestrial agamids (Figure 1b,c). We did not reveal other significant differences in the structure of the vertebrae of autotomic and regenerating caudal appendages and vertebrae of agamids incapable of regeneration, but additional studies are needed here on more extensive data.

**Figure 2:**
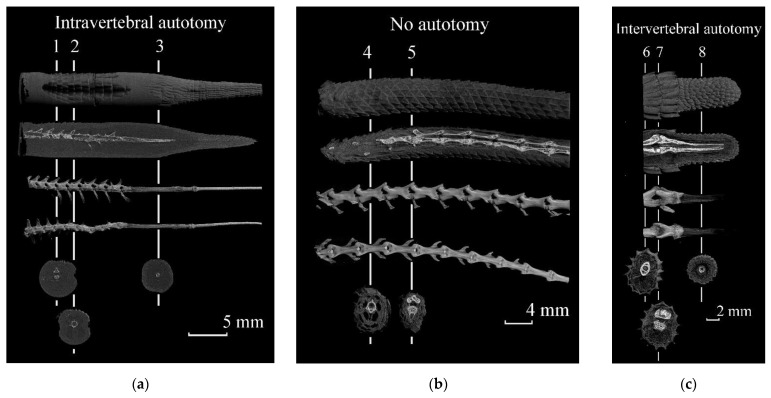
Three-dimensional micro-CT reconstruction of the tails with different combinations of caudal autotomy and regeneration: (**a**) the tail of *Lacerta agilis* (ZISP 31549) with intravertebral autotomy and regeneration—1. section between two intact vertebrae; 2. section through the cartilaginous area dividing the vertebra into two parts and serving as the plane of autotomy; 3. section through a calcified cartilaginous tube. (**b**) the structure of the intact tail of *Trapelus sanguinolentus* (ZISP 31548), incapable of tail ejection—4. section through the middle of the intact tail; 5. section between two intact vertebrae. (**c**) tail of *Paralaudakia caucasia* (ZMMSU 15396) with intervertebral autotomy and regeneration—6. section through the distal region of the intact vertebra; 7. section between two vertebrae, more than half of the second vertebra preceding the plane of autotomy, underwent ablation; 8. section through a calcified cartilage tube. The figure shows (from top to bottom): 3D reconstruction of the tail, scales covering the regenerate are much smaller in size, irregular in shape and in an irregular arrangement; a cut through the sagittal plane, showing the internal structure of the vertebrae and cartilaginous tube, the location of the muscles; 3D reconstruction of the tail vertebrae in the sagittal strip, showing differences in the structure of the intact region of the tail and regenerate; 3D reconstruction of the caudal vertebrae in the frontal plane; slices in the segmental plane. The white vertical lines show the areas of the slices in the segmental plane.

Many species of lizards, after wound healing at the site of urotomy, are able to form a regenerate, which anatomically and morphologically differs from an intact tail (Figure 2). The scales covering the regenerate are much smaller, have an irregular form and no regular arrangement [19], muscle fibers are less structured, there are higher numbers of radial connective tissue septa and the cartilaginous tube that functionally replaces the vertebrae can calcify, but remains for life. The possibility of an autotomic tail rupture at the regenerated site is debatable [35]. A tail bifurcation on the regenerate often indicates that the tail can be broken in this part. However, recent research has shown that bifurcation can occur in non-autotomic species [36] too. They demonstrate that an abnormal regeneration can occur in lizards with intra-vertebral autotomy, inter-vertebral autotomy and in some species that have lost the ability to autotomy. This fact may indicate that autotomy and regeneration can be considered as phenomena that evolved independently of each other. Autotomy was lost secondarily by some lizards for ecological/ethological reasons, but a regeneration as a developmental program remained.

**Figure 3:**
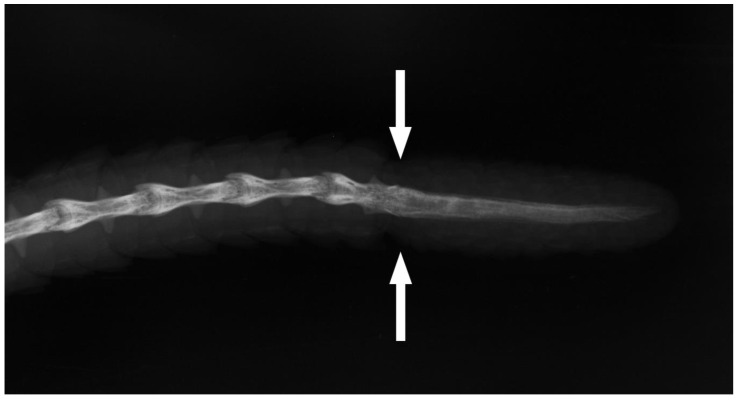
X-ray photograph of *Paralaudakia caucasia* ZISP 19718 (Turkmenistan) with the pseudoautotomy in the distal part of the tail. The partial ablation at the site of the autotomy and the formation of the cartilaginous tube are shown.

Recent agamids demonstrated the following combinations of urotomy and regeneration: (1) pseudoautotomy followed by regenerate formation; (2) pseudoautotomy without regeneration; (3) lack of the ability to urotomy. In some species of agamid lizards, after the tail has been broken, a fragment of the vertebra is visible from the wound. In museum material, we observed this condition in *Leiolepis guttata, Stellagama stellio, Laudakia nupta*, *Laudakia tuberculata*, *Paralaudakia caucasia*, *Mantheyus phuwuanensis*, *Otocryptis wiegmanni* and *Calotes versicolor*. Subsequently, the distal part of such a vertebra underwent partial ablation, which apparently promotes wound healing. After the formation of the regenerate, on X-ray images, such a tail resembled that of a truly autotomic lizard (Figure 3) and this can mislead the researcher, so such conditions should be carefully examined. We summarized information from the literature, supplemented by our material on the variation in the ability for pseudoautotomy and postautotomic regeneration in all the evolutionary lineages [37,38] of agamids (Table 3). The subfamily Uromastycinae with two genera—*Uromastyx* Gray, and *Saara* Gray, 1845 [25]—is most probably not characterized by autotomy and regeneration [2,5,6]. We had no recent available data about this phenomenon after the note of Bellairs and Bryant [2] (p. 330). They wrote that no intravertebral fracture planes were reported in any agamids except in *Uromastyx* “in which traces of an obliterated plane have reputedly been observed”. Arnold [5] indicated that *Uromastyx aegyptia* and *U. thomasi* lose the ability to autotomize during ontogeny. Among the subfamily Amphibolurinae, autotomy is normally restricted to the distal parts of the tail, as was reported for some species of *Amphibolurus* and *Ctenophorus: C. cristatus, C. isolepis, C. maculatus and C. pictus* [5]. This author provided no information on the development of the regenerate. These species are known or suspected to be bipedal; thus, the tail plays an important role in the locomotion of the lizard. Other representatives of Amphibolurinae studied by us (*Gowidon longirostris*, *Hypsilurus bruijnii*, *H. modestus*, *Pogona barbata*, *P. vitticeps*) had intact tails, but the limited sample size did not allow reliable conclusions. The formation of regenerates was recorded in *Ctenophorus caudicinctus, Tropicagama temporalis, Intellagama lesueurii*, but not in *Diporiphora* (Table 3). According to our data, at least four of nine species of the genus *Leiolepis* (Leiolepidinae)—*L. belliana*, *L. guentherpetersi*, *L. guttata* and *L. reevesii*—can form a regenerate after pseudoautotomy.

For the lizards of the subfamily Agaminae, three states of pseudoautotomy and regeneration are known. The first one is the pseudoautotomy followed by the formation of a regenerate (genus *Acanthocercus*, some species of the genera *Agama* (*A. agama*), *Laudakia* (*L. agrorensis*, *L. melanura*, *L. nupta* and *L. tuberculata*), *Paralaudakia* (*P. caucasia*, *P. erythrogaster*, *P. himalayana*, *P. lehmanni* and *P. microlepis*), *Pseudotrapelus* (*P. sinaita*) and *Stellagama* (*S. stellio*)). The second one is the pseudoautotomy without regeneration recorded by Arnold [5] for *Agama aculeata*, *A. agama gracilimembris*, *A. hartmanni*, *A. hispida*, *A. kirkii*, *A. mossambica*, *A. mwanzae*, *A. persimilis* and *X. batillifera*. The third state is the lack of the ability to urotomy (genera *Phrynocephalus* and *Trapelus*). Among Draconinae, there are only several studied species for which we have reliable evidence for caudal autotomy and regeneration: *Bronchocela cristatella, Coryphophylax subcristatus* and *Mantheyus phuwuanensis*. The remaining species either do not form a regenerate (*Calotes calotes*, *Calotes versicolor*, *Otocryptis wiegmanni*, *Psammophilus*, *Sitana*), or are non-autotomic. For *C. calotes*, *Psammophilus*, the cases of regeneration were registered [24] (Table 3) but there was no confirmation of these facts. In *Gonocephalus chamaeleontinus*, *Pelturagonia nigrilabris*, we observed only the condition of pseudoautotomy, but we failed to record any regenerated tails.

Thus, the subfamilies Uromastycinae and, probably, Hydrosaurinae, contain non-autotomic species, and Leiolepidinae include lizards capable of pseudoautotomy and regeneration. Agaminae, Draconinae and Amphibolurinae include species with all combinations of pseudoautotomy and regeneration. There are about 60 genera and 550 species of agamids [25], of which 55 species and 12 genera only were studied; thus, we will have more new facts about the ability/disability to urotomy and regenerate within this family. Arnold [5] underlined that intervertebral autotomy is the derived condition in comparison with the non-autotomic state in Acrodonta and may contain a relatively slight modification. He noted that in agamid lizards, it is mostly known in association with two ecomorphological types: (1) Climbing mostly petrophilic rocky forms (*Agama*, *Pseudotrapelus*, *Laudakia*, *Paralaudakia*, *Stellagama* and *Psammophilus*). (2) Terrestrial forms climbing in vegetation with relatively long hind legs and very long tails that become extremely slender distally (*Bronchocela*). Intervertebral autotomy as the derived state compared with the primitive state of the absence of autotomy in agamids may have arisen many times in *Physignathus*, *Diporiphora*, the lineage containing *Amphibolurus* and *Lophognathus*; *Psammophilus*, the *Otocryptis-Sitana* lineage and subgeneric groupings of *Agama* s. lat. (*Agama* s. strict., *Pseudotrapelus* and *Stellagama*, *Laudakia*, *Paralaudakia*, *Xenagama*). An outgroup comparison also confirmed the condition is derived, as they do for colubrid snakes.

**Figure 4:**
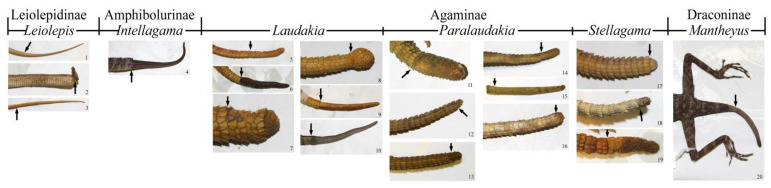
Different types of tail regenerates of agamid lizards of different subfamilies. 1. *Leiolepis belliana* (China, ZISP 17166); 2. *Leiolepis guentherpetersi* (Vietnam, ZISP 21877); 3. *Leiolepis guttata* (Vietnam, ZISP 20354); 4. *Intellagama lesueurii* (Australia, ZISP 20455); 5. *Laudakia nupta* (Persia (Iran), ZISP 23890); 6. *Laudakia nupta* ((Iran, ZISP 11403); 7. *Laudakia nupta* (Iran), ZISP 11402); 8. *Laudakia nupta* (Iran, ZISP 11399); 9. *Laudakia nupta* (Iran, ZISP 11397); 10. *Laudakia tuberculata* (Nepal, ZISP 20424); 11. *Paralaudakia caucasia* (Iran, ZISP 10426); 12. *Paralaudakia caucasia* (Turkmenistan, ZMMSU R-15396); 13. *Paralaudakia stoliczkana* (Mongolia, ZMMSU R-5740); 14. *Paralaudakia erythrogaster* (Iran, ZMMSU R-13517); 15. *Paralaudakia himalayana* (Kirgizstan, ZMMSU 251); 16. *Paralaudakia lehmanni* (Uzbekistan, ZISP 20117); 17. *Stellagama stellio* ((Iran, ZISP 23892); 18. *Stellagama stellio* (Turkey, ZISP 23479); 19. *Stellagama stellio* (Israel, ZISP 22065); 20. *Mantheyus phuwuanensis* (Laos, ZISP 30629). For a more detailed description of the types of tail regenerates in *Paralaudakia* agamids, see here [20].

The Agamidae family is remarkable in that it includes species forming the regenerates of unusual shapes. We supplemented the available information on the morphology of regenerates [23] based also on an examination of the museum specimens of *Paralaudakia* [20] and identified six types of regenerate’s characteristic of the studied group: knob-shaped jagged, knob-shaped smooth, conical jagged, conical smooth, club-shaped and narrowed. Some types of *Paralaudakia* regenerates, complemented by variations in other subfamilies, are presented (Figure 4).

**Figure 5:**
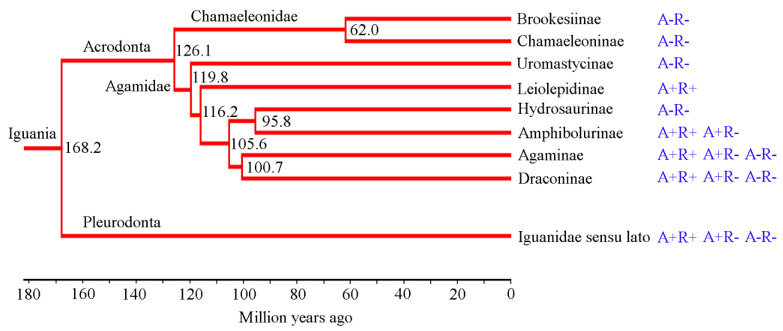
Phylogenetic tree of Iguania (combining phylogenomic and supermatrix approaches based on 52 genes and 4162 species for squamate reptiles) with a time calibration and indications of different types of autotomy and regeneration (after Zheng and Wiens [47] with modifications).

One of the most important aspects of urotomy research is the phylogenetic transformation of the ability to shed the tail. According to modern phylogenetic views on iguanomorphic lizards, Agamidae, together with Chamaeleonidae, form the Acrodonta clade, sister to the pleurodont Iguanidae sensu lato [38,47,48]. Chamaeleonidae, which are mainly arboreal and the sister group to the rest of Iguania, include only non-autotomic species. According to the recent studies of Zheng and Wiens [47], combining the phylogenomic and supermatrix approaches, the radiation of Agamidae of non-autotomic Uromastycinae and other Agamidae occurred about 119.8 million years ago (Figure 5). The autotomic and forming regenerated the Leiolepidinae lineage separated from this group after 3.6 million years (116.2 million years ago). Two lineages appeared after another 10.6 million years (105.6 million years ago). The first lineage probably included non-autotomic Hydrosaurinae and autotomic Amphibolurinae (diverged 95.8 million years ago), some of which can develop regenerates, while others do not. The second phylogenetic lineage diverged 100.7 million years ago led to the separation of Agaminae and Draconinae. Both of the latter groups include species capable of pseudoautotomy and regeneration. Draconinae includes *Bronchocela cristatella* and *M. phuwuanensis* (a sister species to the other of Draconinae), shedding the tail and, subsequently, regenerating it, and many non-autotomic species.

The most simple explanation for the lack of the ability for pseudoautotomy in Chamaeleonidae (a sister group for Iguania within Acrodona) and Uromastycinae, which is a sister group for the remaining Agamidae, is associated with the origin of iguanomorphic lizards from non-autotomic Prolacertilia. On the other hand, an in-depth analysis of urotomy cases in reptiles showed that the presence of a true autotomy was a plesiomorphic state in relation to other variants of urotomy [5]. According to Arnold [5], the sequence of evolutionary changes is as follows: intravertebral autotomy, no autotomy, intervertebral autotomy. If this assumption is correct, then Prolacertilia is a group of lizards with true autotomy, the ability for which was obtained by the more advanced Scinco-Gekkonomorphas.

Early iguanomorphic lizards, primitive in relation to Scinco-Gekkonomorpha, lost the ability to urotomy, and this state was preserved in Chamaeleonidae and early Agamidae (Uromastycinae). A further divergence of the phylogenetic lineages of agamids led to the restoration of autotomy by some groups as a pseudoautotomy.

Recent reconstructions of the phylogeny of squamates based on molecular genetic data differed significantly from morphological ones. This was especially true for iguanomorphic lizards, which are characterized by a number of primitive traits, but occupy a position among advanced forms on such molecular trees [22,38]. Nevertheless, the sequence of evolutionary changes in the ability for urotomy was preserved: phylogenetic lineages of squamates with autotomy planes in the vertebral body, as a rule, are the sister groups.

The terminal group in many molecular phylogenies is snakes, most of which have lost the ability to urotomy; only in a few species it has been restored in the form of pseudoautotomy. The list of such species is given in a review of Crnobrnja-Isailovic et al. [12] and includes the members of two families: Colubridae (*Hierophis viridiflavus*, *Dolichophis caspius*, *Hemorrhois hippocrepis*, *Natrix tessellata*, *Nerodia erythrogaster*, *N. sipedon*, *Thamnophis sauritus*, *T. sirtalis*, *Dendrophidion dendrophis*, *Drymoluber brazili*, *D. dichrous*, *Mastigodryas boddaerti*, *Pliocercus elapoides*, *Scaphiodontophis* sp., *Urotheca* sp., *Natriciteres variegata*, *Amphiesma stolata*, *Sibynophis* sp., *Xenochrophis piscator*) and Lamprophiidae (*Psammophis phillipsii*). Paleontological data [49] considered snakes as originated from mosasaurs and other varanoid lizards or burrowing lizards with reduced limbs. When swimming, lizards actively use their tail, but not their limbs. Then, the loss of even an insignificant part of the tail during autotomy can lead to significant difficulties in movement and a decrease in viability. In addition, Varanids are a group of lizards that have completely lost the ability to urotomy. In such a context, the origin of snakes from this group is quite consistent with the concept of phylogenetic transformations of the ability to autotomy in squamate reptiles. Snakes inherited this condition, and some Colubridae re-established the urotomy in the form of a non-specialized pseudoautotomy.

According to the other theory of the origin of snakes, Scincoidea, the ancestors of Amphisbaenia (or common ancestors of Amphisbaenia and Dibamidae), as well as the family of Gekkota (Pygopodidae), are considered as the hypothesized ancestors [49]. All these groups include species with an intravertebral autotomy, i.e., a true autotomy. Some, for example, members of the Trogonophidae family, have lost the ability to regenerate, and the fracture plane contains only one vertebra [5]. In this case, the reconstruction of the phylogenetic transformations of the caudal vertebrae, which led to the loss of urotomy, is significantly complicated, since groups important for understanding this process (Amphisbaenia and Dibamidae) occupy a controversial taxonomic position in the Squamata system, and the location of autotomic and non-autotomic groups on the phylogenetic tree seems not to be natural.

Large-scale molecular studies of the mitochondrial and nuclear genomes of squamate reptiles cardinally changed the existing views on the relationships of these species [26,50]. The available results suggest that Iguania now is not the basal clade of squamates but, on the contrary, is regarded as a derived evolutionary lineage clustering with anguinomorphan lizards and/or snakes. Subsequent molecular studies have confirmed that the group Scincomorpha is not monophyletic, and they demonstrated profound differences in the interpretation of the position of Iguania as a sister group to the snakes [51], Anguimorpha [26,52,53], Gekkota [54], Anguimorpha + Scincidae [55], or Anguimorpha + Serpentes [51]. In the recovered phylogenetic tree [56], Iguania is not a monophyletic group, while Acrodonta and Serpentes form a clade positioned as a sister group relative to the remaining squamates; Iguanidae is united in one clade with Scincomorpha. According to this new phylogenetic scheme, neither monitor lizards nor fossorials (amphisbaenas and dibamids) are regarded as intermediate relatives of snakes. However, some researchers assert that molecular data cannot be regarded as the only true means to resolve the phylogeny because of the inadequate interpretation of a large genetic distance between the squamate reptiles and their only close extant-related taxon, Rhynchocephalia, with its only living species, tuatara *Sphenodon punctatus* [57]. These authors infer that the molecular data support the traditional morphological phylogenetic hypothesis on the monophyletic group of iguanian lizards, which is a sister lineage relative to the remaining squamates. Despite numerous publications on this issue [27,28,29], the discussion is still in progress, as is the search for new productive approaches to solve this problem [30,57,58].

Another interesting and unresolved issue is related to the morphological transformations of the axial skeleton, in particular its caudal region, which led to the appearance of a fracture plane in the vertebral body. The great morphological diversity of the caudal vertebrae in squamate reptiles, the unstable number of vertebrae within taxonomic groups and even in different populations of the same species as well as ontogenetic transformations (loss of fracture planes in some Iguanidae sensu lato) significantly complicates the solution of this problem. The most likely type of vertebra in early reptiles is similar to the gastrocoelic vertebra in amphibians. Evolutionarily, it develops from the embolomeric aspect because of the strengthening of the pleurocenter and the reduction in the hypocenter. The hypocenter loses its connection with the neural arch and, in the form of a sickle-shaped body (intercenter), lies at the bottom of the vertebra [59]. Intercenters in the caudal vertebrae, presumably, were transformed into hemapophysis [60], and the body of the caudal vertebra (pleurocenter) increased longitudinally. It is highly likely that the complicated, multicomponent structure of the vertebra (hypocenter, pleurocenter, neural arch) and their independent ossification determined the possibility of forming a fracture plane and an intravertebral autotomy. Since the pleurocenter in the caudal vertebra is transformed into a hemapophysis, it cannot participate in the formation of the anterior component of the vertebral body, which remains in the tail of the lizard after a true autotomy. To identify the cause of the “double” structure of the vertebrae of recent lizards with true autotomy, it is necessary to continue the deep analysis of the paleontological data and embryological studies of recent lizards of different taxonomic groups, not only model species.

## 4. Conclusions

Despite the long history of studying the phenomenon of urotomy in reptiles, a wide range of issues in this area remains unresolved, including methodological reasons. Iguanomorpha lizards and, in particular, Agamidae are the most promising group in this respect, whose studies are still limited to a small number of species. Recent agamids include species with the different combinations of urotomy and regeneration: 1. pseudoautotomy followed by regenerate formation; 2. pseudoautotomy without regenerate development; 3. lack of ability to urotomy. The further accumulation of the new data is necessary to continue for the integrative analysis in the morphological, paleontological and embryological studies of recent lizards of different taxonomic groups, to identify the stages of evolution of the ability to autotomy and regeneration. This will provide progress in this field.

## Figures and Tables

**Table 1:** The number of analyzed museum specimens in the herpetological collections of the ZISP RAS and ZMMSU (sample size).

Genus	Species	Total Number of Examined Specimens	Number of Specimens with Uncertain Cases of Broken Tails	Number of Specimens with Autotomized and Regenerated Tails
Leiolepidinae				
*Leiolepis*	*L. belliana*	4	0	4
	*L. guentherpetersi*	3	0	3
	*L. guttata*	10	2	8
	*L. reevesii*	8	2	6
Amphibolurinae				
*Gowidon*	*G. longirostris*	1	0	1
*Hypsilurus*	*H. bruijnii*	1	0	1
	*H. modestus*	5	0	5
*Intellagama*	*I. lesueurii*	5	4	1
*Lophosaurus*	*L. spinipes*	3	0	3
*Physignathus*	*P. cocincinus*	6	0	6
*Pogona*	*P. barbata*	1	0	1
	*P. vitticeps*	2	0	2
Agaminae				
*Laudakia*	*L. nupta*	88	36	52
	*L. tuberculata*	9	1	8
*Paralaudakia*	*P. caucasia*	246	9	237
	*P. erythrogaster*	32	4	28
	*P. himalayana*	57	12	45
	*P. lehmanni*	220	37	183
	*P. microlepis*	6	1	5
	*P. stoliczkana*	132	15	117
*Stellagama*	*S. stellio*	67	1	66
Draconinae				
*Calotes*	*C. calotes*	8	1	7
	*C. versicolor*	59	2	57
*Gonocephalus*	*G. chamaeleontinus*	9	2	7
	*G. liogaster*	6	0	6
	*G. sophiae*	1	0	1
*Malayodracon*	*M. robinsonii*	2	0	2
*Mantheyus*	*M. phuwuanensis*	4	1	3
*Otocryptis*	*O. wiegmanni*	3	0	3

**Table 2:** Incidence of pseudoautotomy of studied agamid lizards.

Species	Sample Size	Intact Tails		Pseudoautotomy					
				In Distal Third of Tail		In the Middle of Tail		In Proximal Third of Tail	
		n	%	n	%	n	%	n	%
Leiolepidinae									
*Leiolepis*:									
*L. belliana*	4	2	50.0	1	25.0	0	0.0	1	25.0
*L. guentherpetersi*	3	2	66.7	1	33.3	0	0.0	0	0.0
*L. guttata*	8	4	50.0	1	12.5	3	37.5	0	0.0
*L. reevesii*	6	4	66.6	1	16.7	1	16.7	0	0.0
Amphibolurinae									
*Gowidon longirostris*	1	1	100.0	0	0.0	0	0.0	0	0.0
*Hypsilurus*:									
*H. bruijnii*	1	1	100.0	0	0.0	0	0.0	0	0.0
*H. modestus*	5	5	100.0	0	0.0	0	0.0	0	0.0
*Intellagama lesueurii*	1	0	0.0	1	100.0	0	0.0	0	0.0
*Lophosaurus spinipes*	3	2	66.7	1	33.3	0	0.0	0	0.0
*Physignathus cocincinus*	6	4	66.7	2	33.3	0	0.0	0	0.0
*Pogona*:									
*P. barbata*	1	1	100.0	0	0.0	0	0.0	0	0.0
*P. vitticeps*	2	2	100.0	0	0.0	0	0.0	0	0.0
Agaminae									
*Laudakia*:									
*L. nupta*	52	34	65.4	11	21.2	6	11.5	1	1.9
*L. tuberculata*	8	4	50.0	2	25.0	1	12.5	1	12.5
*Paralaudakia*:									
*P. caucasia*	237	148	62.4	72	30.4	12	5.1	5	2.1
*P. erythrogaster*	28	15	53.6	10	35.7	1	3.6	2	7.1
*P. himalayana*	45	30	66.7	9	20.0	5	11.1	1	2.2
*P. lehmanni*	183	159	86.9	21	11.5	3	1.6	0	0.0
*P. microlepis*	5	2	40.0	3	60.0	0	0.0	0	0.0
*P. stoliczkana*	117	85	72.6	32	27.4	0	0.0	0	0.0
*Stellagama stellio*	66	43	65.2	21	31.8	2	3.0	0	0.0
Draconinae									
*Calotes*:									
*C. calotes*	7	6	85.7	1	14.3	0	0.0	0	0.0
*C. versicolor*	57	46	80.7	6	10.5	3	5.3	2	3.5
*Gonocephalus*:									
*G. chamaeleontinus*	7	3	42.9	4	57.1	0	0.0	0	0.0
*G. liogaster*	6	5	83.3	1	16.7	0	0.0	0	0.0
*G. sophiae*	1	1	100.0	0	0.0	0	0.0	0	0.0
*Malayodracon robinsonii*	2	2	100.0	0	0.0	0	0.0	0	0.0
*Mantheyus phuwuanensis*	3	0	0.0	1	33.3	1	33.3	1	33.3
*Otocryptis wiegmanni*	3	1	33.3	2	66.7	0	0.0	0	0.0

**Table 3:** Incidence of broken and regenerated tails among agamid lizards.

Species	Presence of Autotomy and Regeneration ^1^	Source
Uromastycinae		
Genus *Uromastyx*	A+R?	[2]
*Uromastyx aegyptia*	A-R-	[5]
*Uromastyx thomasi*	A-R-	[5]
Leiolepidinae		
*Leiolepis belliana*	A+R+	(Figure A1a and Figure S7a)
*Leiolepis guentherpetersi*	A+R+	(Figure A1b and Figure S7b)
*Leiolepis guttata*	A+R+	Figure A1c and Figure S7c)
*Leiolepis reevesii*	A+R+	(Figure A1d and Figure S7d)
Hydrosaurinae		
Genus *Hydrosaurus*	A-R-	Probably
Amphibolurinae		
*Amphibolurus*	A+R?	Some species of genus [5]
Genus *Ctenophorus*		
*Ctenophorus caudicinctus*	A+R+	[5]
*Ctenophorus cristatus*	A+R-?	[5]
*Amphibolurus* (*Ctenophorus*) *decresii*	A+R+	[24]
*Ctenophorus isolepis*	A+R-?	[5]
*Ctenophorus maculatus*	A+R-?	[5]
*Ctenophorus pictus*	A+R-?	[5]
Genus *Diporiphora*	A+R-	[5,6]
*Diporiphora bilineata*	A+R-	[5]
*Intellagama lesueurii*	A+R+	[5,39], (Figure A1e and Figure S7e)
*Lophognathus gilberti*	A+R-	[6]
*Lophosaurus spinipes*	A+R?	(Figure A1f and Figure S7f)
*Physignathus cocincinus*	A+R?	(Figure A1g and Figure S7g)
*Tropicagama temporalis*	A+R+	[5,6]
Agaminae		
Genus *Acanthocercus*	A+R+	[5]
Genus *Agama*	A+R+	[5]
*Agama agama*	A+R+	[5,23]
*Agama aculeata*	A+R-?	[5]
*Agama anchietae*	A+R-?	[5]
*Agama atra*	A+R+	[5]
*Agama benueensis* (=*Agama doriae*)	A+R+	[5]
*Agama boueti*	A+R+	[5]
*Agama caudospinosa*	A+R+	[5]
*Agama distanti* (=*Agama aculeata*)	A+R-?	[5]
*Agama doriae*	A+R+	[5]
*Agama gracilimembris*	A+R-	[5]
*Agama hartmanni*	A+R-?	[5]
*Agama hispida*	A+R-?	[5]
*Agama kirkii*	A+R-?	[5]
*Agama mossambica*	A+R-?	[5]
*Agama mwanzae*	A+R-?	[5]
*Agama paragama*	A+R+	[5]
*Agama persimilis*	A+R-?	[5]
*Agama picticauda*	A+R+	[40]
*Agama planiceps*	A+R+	[5]
*Agama rueppelli*	A+R+	[5]
*Agama sankaranica*	A+R+	[5]
*Agama spinosa*	A+R+	[5]
*Agama sylvanus* (=*Agama africana*)	A+R+	[5]
*Agama weidholzi*	A+R-?	[5]
*Acanthocercus adramitanus*	A+R+	[5]
*Acanthocercus annectans*	A+R+	[5]
*Acanthocercus atricollis*	A+R+	[5]
*Acanthocercus cyanogaster*	A+R+	[5]
*Acanthocercus phillipsii*	A+R+	[5]
*Acanthocercus yemensis*		[5]
*Laudakia agrorensis*	A+R+	[5]
*Laudakia melanura*	A+R+	[5]
*Laudakia nupta*	A+R+	[5,20], (Figure A1h and Figure S7h)
*Laudakia tuberculata*	A+R+	[5,24], (Figure A1i and Figure S7i)
*Paralaudakia caucasia*	A+R+	[5,20,41], (Figure A1j and Figure S7j)
*Paralaudakia erythrogaster*	A+R+	[5,20], (Figure A1k and Figure S7k)
*Paralaudakia himalayana*	A+R+	[5,20,24], (Figure A1l and Figure S7l)
*Paralaudakia lehmanni*	A+R+	[5,20], (Figure A1m and Figure S7m)
*Paralaudakia microlepis*	A+R+	[5,20], (Figure A1n and Figure S7n)
*Paralaudakia stoliczkana*	A+R+	[5,20], (Figure A1o and Figure S7o)
*Phrynocephalus*	A-R-	[5]
*Pseudotrapelus*	A-R-	[5]
*Pseudotrapelus sinaitus*	A+R+	[5]
*Stellagama stellio*	A+R+	[5,6,10,23], (Figure A1p and Figure S7p)
*Trapelus*	A-R-	[5,6]
*Xenagama batillifera*	A+R-	[2,5]
Draconinae		
*Bronchocela cristatella*	A+R+	[42]
*Calotes calotes*	A+R-A-R-	[43,44], (Figure A1q and Figure S7q)
*C. ophiomachus* (*C.calotes*)	A+R+	[24]
*C. mystaceus*	A+R	[24]
*C. versicolor*	A+R-	[10], (Figure A1r and Figure S7r)
Genus *Diploderma*		[5]
*Diploderma ngoclinense*	A+R?	[45]
Genus *Gonyocephalus*		[5]
*Gonyocephalus subcristatus* (=*Coryphophylax subcristatus*)	A+R+	[2,24,42]
*Gonocephalus chamaeleontinus*	A+R-?	(Figure A1s and Figure S7s)
*Gonocephalus liogaster*	A+R?	(Figure A1t and Figure S7t)
*Mantheyus phuwuanensis*	A+R+	[46], (Figure A1u and Figure S7u)
*Otocryptis*	A+R-	[5]
*Otocryptis wiegmanni*	A+R-	[5], (Figure A1v and Figure S7v)
*Pelturagonia nigrilabris*	A+R-?	(Figure A1w and Figure S7w)
*Psammophilus*	A+R-	[6]
*Psammophilus dorsalis*	A+R-?	[5]
*Charasia* (=*Psammophilus) blanfordiana*	A+R+	[24]
*Sitana*	A+R-	[6]
*Sitana ponticeriana*	A+R-	[5,6]

^1^ A+R+ pseudoautotomy followed by regenerate formation; A+R- pseudoautotomy without regenerate formation; A-R- lack of ability to urotomy; A?—no convincing evidence of pseudoautotomy; R?—no convincing evidence of regenerate formation.

**Figure A1:**
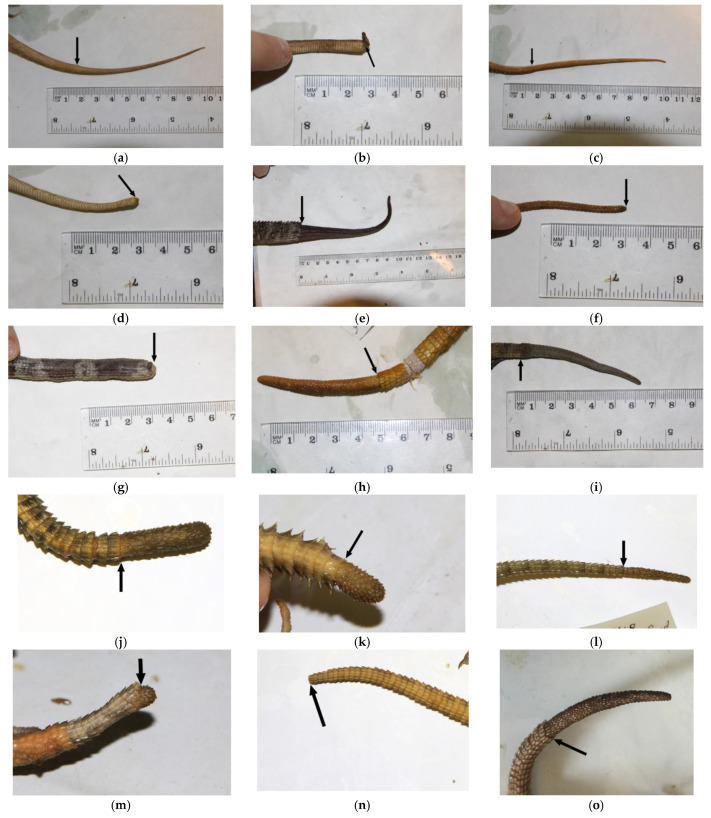

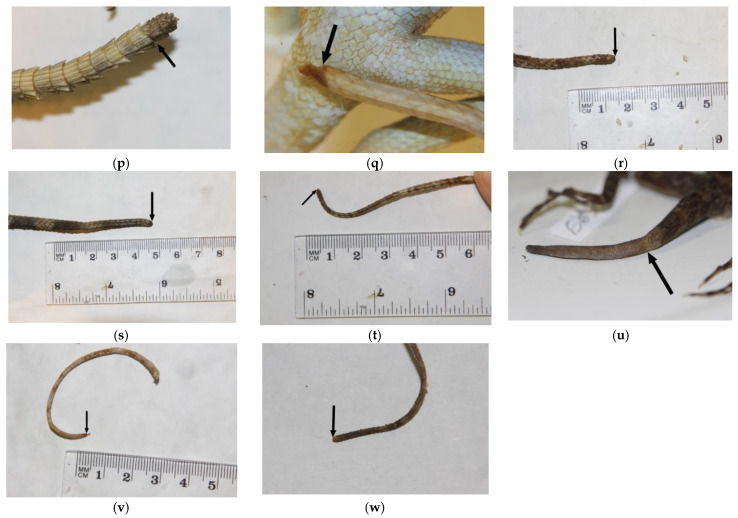
Fragments of the tail, demonstrating cases of its loss and the formation of regenerate (if any) in agamid lizards: (**a**) *Leiolepis belliana* (ZISP 17166); (**b**) *Leiolepis guentherpetersi* (ZISP 21877); (**c**) *Leiolepis guttata* (ZISP 20354); (**d**) *Leiolepis reevesii* (ZISP 20355); (**e**) *Intellagama lesueurii* (ZISP 20455); (**f**) *Lophosaurus spinipes* (ZISP 20466); (**g**) *Physignathus cocincinus* (ZISP 22567); (**h**) *Laudakia nupta* (ZISP 11397); (**i**) *Laudakia tuberculata* (ZISP 20424); (**j**) *Paralaudakia caucasia* (ZMMSU R-7308); (**k**) *Paralaudakia erythrogaster* (ZISP 18844); (**l**) *Paralaudakia himalayana* (ZMMSU R-9151); (**m**) *Paralaudakia lehmanni* (ZISP 19964); (**n**) *Paralaudakia microlepis* (ZMMSU R-11916); (**o**) *Paralaudakia stoliczkana* (ZISP 20220); (**p**) *Stellagama stellio* (ZISP 23479); (**q**) *Calotes calotes* (ZISP 935); (**r**) *Calotes versicolor* (ZISP 20237); (**s**) *Gonocephalus chamaeleontinus* (ZISP 2469); (**t**) *Gonocephalus liogaster* (ZISP 18573); (**u**) *Mantheyus phuwuanensis* (ZISP 30629); (**v**) Otocryptis wiegmanni (ZISP 3420); (**w**) Pelturagonia nigrilabris (ZISP 18546). Arrows show the plane of autotomy.

## Data Availability

Not applicable.

## Supplementary Materials

The following are available online at https://cloud.mail.ru/public/vHYz/RYLZSwMkL, Figure S1: Micro-CT data *Intellagama lesueurii*, Figure S2: Micro-CT data *Paralaudakia caucasia*, Figure S3: Micro-CT data *Trapelus sanguinolentus*, Figure S4: Micro-CT data *Mantheyus phuwuanensis*, Figure S5: Micro-CT data *Calotes versicolor*, Figure S6: Micro-CT data *Lacerta agilis*, Figure S7: Images of agamas with broken tails and regenerates; the arrow indicates the sites of autotomy.
